# Effect of Age-Related Cartilage Turnover on Serum C-Telopeptide of Collagen Type II and Osteocalcin Levels in Growing Rabbits with and without Surgically Induced Osteoarthritis

**DOI:** 10.1155/2014/284784

**Published:** 2014-03-05

**Authors:** Chung-Cheng Huang, Chen-Chang Lee, Ching-Jen Wang, Feng-Sheng Wang, Hsuan-Ying Huang, Shu-Hang Ng, Chia-Yi Tseng, Sheung-Fat Ko

**Affiliations:** ^1^Department of Radiology, Kaohsiung Chang Gung Memorial Hospital, Chang Gung University College of Medicine, 123 Ta-Pei Road, Niao-Sung Hsiang, Kaohsiung 833, Taiwan; ^2^Department of Orthopedic Surgery, Kaohsiung Chang Gung Memorial Hospital, Chang Gung University College of Medicine, 123 Ta-Pei Road, Niao-Sung Hsiang, Kaohsiung 833, Taiwan; ^3^Department of Medical Research, Kaohsiung Chang Gung Memorial Hospital, Chang Gung University College of Medicine, 123 Ta-Pei Road, Niao-Sung Hsiang, Kaohsiung 833, Taiwan; ^4^Department of Pathology, Kaohsiung Chang Gung Memorial Hospital, Chang Gung University College of Medicine, 123 Ta-Pei Road, Niao-Sung Hsiang, Kaohsiung 833, Taiwan

## Abstract

This study aims to determine the effect of age-related cartilage turnover on the serum C-telopeptide of type II collagen (CTX-II) and osteocalcin (OC) levels in growing rabbits with and without surgically induced osteoarthritis. Twenty-four New Zealand male 3-month-old rabbits were randomized into three operated groups (*n* = 6 per group, with surgically induced osteroarthritis in the right knee; after blood sampling, the knees were harvested following euthanization at 2, 3, and 6 months after surgery) and a control group (*n* = 6, blood samples were obtained monthly between 3 and 15 months). Histomorphologically, the medial femoral condyles, particularly the central parts, harbored the most severe osteoarthritic changes among the operated rabbits. The serum levels of CTX-II and OC decreased in the controls from 3 to 11 months and then remained stable. No significant differences in the serum CTX-II and OC levels between the osteoarthritic rabbits and controls were observed. The osteoarthritic-to-normal ratios (ONRs, the ratios of serum CTX-II or OC levels in osteoarthritic rabbits to those of the controls at same ages) enabled an overall assessment of osteoarthritis and age-related cartilage turnover. Elevated CTX-II ONRs were observed in rabbits with mild to advanced osteoarthritis. However, the OC ONRs were unhelpful in assessing osteoarthritic growing rabbits.

## 1. Introduction

Osteoarthritis is a chronic debilitating disease characterized by progressive damage of articular cartilage and subchondral bone with resulting functional impairment and disability [1]. Traditionally, cartilage is indirectly assessed using radiography, and the loss of joint space width is presumed to be due to cartilage thinning. However, radiography is limited by a low reproducibility of identical positioning in serial assessments, lack of direct quantification of cartilage loss, and low sensitivity in detecting subtle cartilage loss [1, 2]. Magnetic resonance imaging (MRI) enables the direct visualization of cartilage, and the most recent technical advances enable high-resolution morphological and compositional assessment of articular cartilage and subchondral bones [3]. In addition, application of MRI biomarkers in predicting clinical outcome and the need of joint replacement have also been addressed [4, 5]. However, MRI is also limited by difficulties with identical repositioning, different MRI systems and pulse sequences in different centers, lack of consensus definitions of osteoarthritis diagnosis and grading, and expensive cost [1].

Recently, the potential use of applying biomarkers in the diagnosis and monitoring of osteoarthritis has been highlighted [6–13]. Osteoarthritic damage results in the loss of two main articular cartilage constituents, proteoglycans and type II collagen [6, 7]. A great variety of biomarkers, such as C- or N-propeptides of procollagen type II; N-propeptides of collagen type I and type III; collagen type II neoepitope; C-telopeptides of collagen type I and type II (CTX-II); matrix metalloproteases; and C-reactive protein, have been previously explored [6–9]. Several reviews on currently investigated biomarkers for assessing collagen metabolism in cartilage or bone, aggrecan metabolism in cartilage, and noncollagenous proteins in inflammation and/or fibrosis have thoroughly addressed the clinical status of these biomarkers [10–13]. Prognostic biomarkers may be used in identifying subgroups of osteoarthritis in whom the disease progresses at different rates, differentiate phenotypes within a heterogeneous knee osteoarthritis population, and facilitate the development of disease-modifying osteoarthritis drugs [11, 12]. Among these biomarkers, CTX-II has been found to be a feasible biomarker that offers early detection of cartilage degradation, harbors a good association with radiographic signs of joint damage, and may determine radiographic subtypes of osteoarthritis in various joints in subject with familial osteoarthritis [7–10, 13–15]. Subchondral bone also plays an important role in the initiation and progression of osteoarthritis [16]. Osteocalcin (OC) is synthesized by osteoblasts for bone formation and is released into the circulating blood during bone turnover [16, 17]. Bone metabolism is activated in early osteoarthritis with enhanced bone mineralization, which may potentially be assessed by determining the serum OC level [18] and serum OC can be used as a biomarker for osteoarthritis of the knee [19].

In addition to enhancing cartilage metabolism in arthritis, Mouritzen et al. have reported that cartilage turnover as assessed by the levels of CTX-II and OC may also be influenced by other factors, including age, menopause, hormone replacement therapy, and body mass index in healthy men and women [20]. Contrastingly, in animal models for preclinical studies on the role of CTX-II in the diagnosis and treatment of arthritis, factors other than cartilage damage have garnered far less concerns [21–23]. Measurements of serum or urine CTX-II have been described as useful biomarkers for early cartilage changes in osteoarthritis in adult rabbits [21, 22]. Contrastingly, the lack of significant differences of CTX-II levels between the surgically induced osteoarthritis and normal controls in young rabbits has also been reported in a 5-month longitudinal study [24]. Nevertheless, the effects of age of the animals on the biomarkers of cartilage turnover and degradation have not been fully addressed. The aim of this study was to clarify the effect of age-related cartilage turnover on the levels of serum CTX-II and OC in healthy rabbits from a young age to adulthood via a 12-month longitudinal observation and a cross-sectional study of the biomarkers and histomorphological features in surgically induced osteoarthritis in young rabbits.

## 2. Materials and Methods

### 2.1. Ethical Statement

All animal experimental procedures were approved by the *Institutional Committee on Animal Care, Use, and Research* and performed according to the Guide for the Care and Use of Laboratory Animals (NIH publication number 85-23, National Academy Press, Washington, DC, USA, revised 1996).

### 2.2. Animals and Experimental Protocol

Twenty-four 3-month-old New Zealand male white rabbits (BioLasco Technology, Taiwan) with a mean body weight of 2.1 kg ± 0.13 kg (standard deviation) were used. The rabbits were randomly divided into four groups (*n* = 6 per group). Osteoarthritis of the right knee was surgically induced in eighteen 3-month-old rabbits using a unilateral anterior cruciate ligament transection and partial medial meniscectomy as previously described [24, 25]. For the cross-sectional study of surgically induced osteoarthritis at different time points, the operated rabbits were divided into 3 groups and then euthanized at the age of 5 months (group O5M, *n* = 6, 2 months after operation), 6 months (group O6M, *n* = 6, 3 months after operation), and 9 months (group O9M, *n* = 6, 6 months after operation). Serial blood samples (5 mL each) were collected monthly from 6 healthy rabbits (normal control) from 3 to 15 months for longitudinal observation of the levels of serum CTX-II and OC from a young age to adulthood. These blood samples were designated as N3M, N4M,… to N15M, indicating that the samples were obtained from the control from month 3, month 4,… to month 15, respectively.

### 2.3. Surgical Procedure

The rabbits were anesthetized using an intramuscular injection of Zoletil 50 (0.5 mL/kg) and Rompun 2% (0.2 mL/kg). The knee was approached through a medial parapatellar incision under sterile conditions. After preparation of the joint capsule, the patella was dislocated laterally and the knee joint was placed in full flexion. Next, the anterior cruciate ligament was transected. The meniscotibial ligament was incised with iris scissors, and the anterior half of the meniscus was released and excised. After homeostasis, the capsule, subcutis, and epidermis were closed layer by layer. After surgery, ketoprofen (0.1/kg) and ampicillin (100 mg/kg) were administered for 3 days. The rabbits were housed individually with free cage activity.

### 2.4. Blood Sampling

To avoid diurnal changes, all fasting blood samples were collected between 9:00 and 10:00 am via the central ear artery from each rabbit. For the healthy animals in the control group, blood samples were obtained monthly from 3 to 15 months. For the animals in the operated groups, blood samples were collected just prior to the rabbits being euthanized at 2, 3, and 6 months after surgical induction of osteoarthritis in the knee. The samples were kept frozen at −70°C within one hour until they were further assayed.

### 2.5. Histomorphological Examination of Operated Knee

Animals in the O5M, O6M, and O9M groups were euthanized at 2, 3, and 3 months after surgical induction of osteoarthritis in the knees. The distal part of the femur and the proximal part of the tibia of the operated knees were dissected and fixed in buffered formalin for 24 hours, followed by decalcification in 20% ethylenediaminetetraacetic acid (EDTA) for 6 weeks. Next, the specimens were dehydrated through a graded alcohol series, cleared with xylene, sectioned (5 mm-thickness) along the sagittal plane of each knee component (medial and lateral femoral condyles, and medial and lateral tibial condyles), and embedded in paraffin. For microscopic assessment, two sections (3 *μ*m-thickness) per knee condyle were cut with a Polycut E microtome (Leica Microsystem, Wetzlar, Germany) along the most severely affected parts of the femoral or tibial condyles, and the sections were stained with Safranin O staining to assess the cartilage matrix.

**Figure 1:**
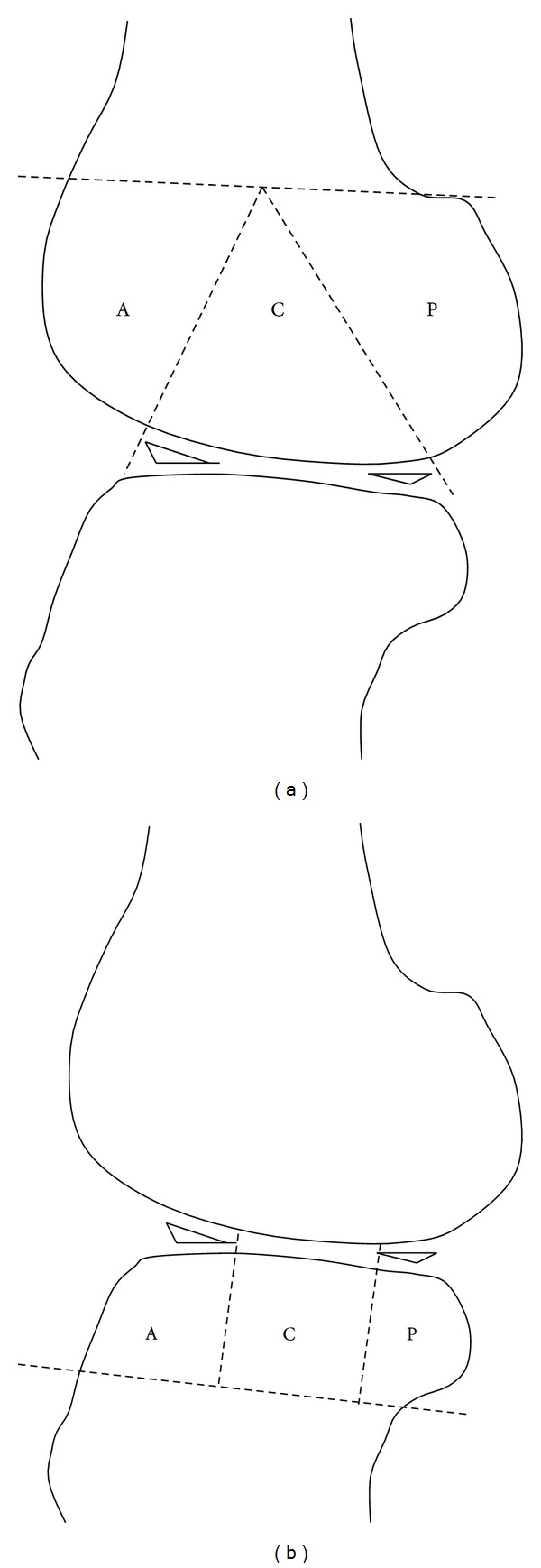
A sketch of the subregions over the femoral (a) and tibial (b) condyles tailored for the measurement of cartilage defect on sagittal histomorphological sections. Both of the femoral and tibial articular surfaces were divided into the anterior (A), central (C), and posterior (P) subregions. Region A of the femur corresponded to the patellofemoral articulation; region C represented the weight-bearing surface, and region P represented the posterior convexity, which articulates only in extreme flexion. Region C of the tibial surface represented the uncovered portion between the anterior and posterior horns of the meniscus centrally.

**Figure 2:**
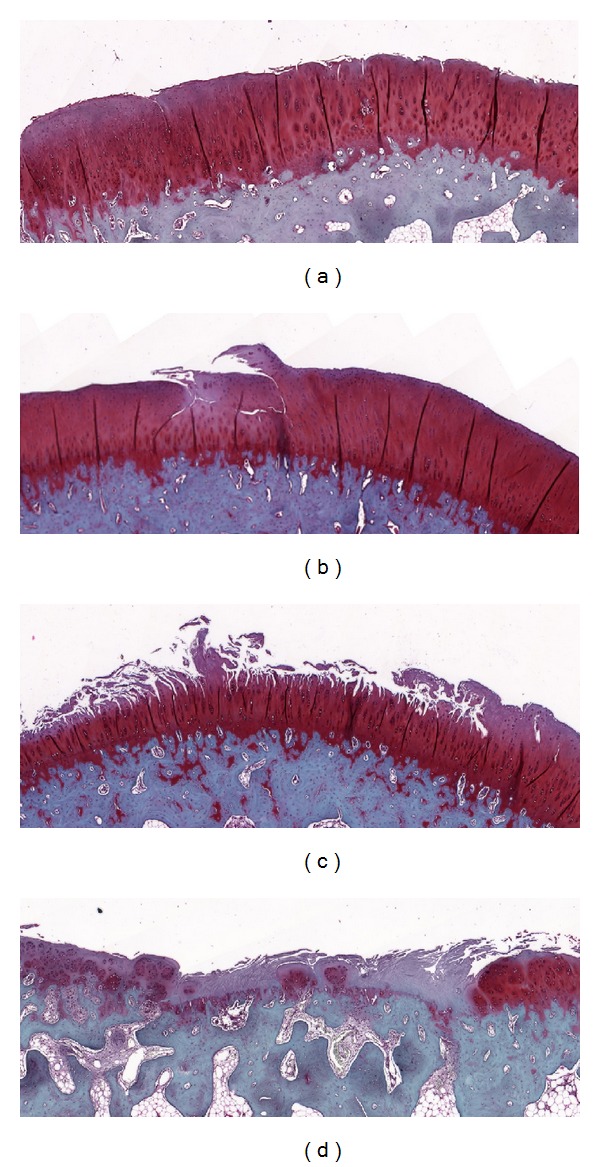
Representative sections of articular osteochondroid tissues stained with Safranin O and magnified at 50x. (a) Grade 3: simple fissure in cartilage with surface abrasion and matrix loss within superficial zone. (b) Grade 3.5: branched fissure extending to the deep zone of cartilage. (c) Grade 4.5: erosion of the cartilage with fibrillated surfaces and mid zone excavation. (d) Grade 5.5: denudation of cartilage with reparative fibrocartilage tissue in bone surface.

On each section, the femoral or tibial condyle was divided into 3 subregions for the assessment of cartilage damage in the anterior, central, and posterior parts (Figure 1). Cartilage damage in each subregion was graded and scored according to the Osteoarthritis Research Society International (OARSI) Osteoarthritis Cartilage Histopathology Assessment System (OOCHAS) [26, 27]: Grade 0, surface and cells intact; Grade 1, surface intact (1.0: edema and/or fibrillation of matrix, proliferation, or hypertrophy of cells; 1.5: cells death); Grade 2, surface discontinuity (2.0: fibrillation through superficial zone; 2.5: surface abrasion with matrix loss within superficial zone); Grade 3, vertical fissures (3.0: simple fissures; 3.5: branched/complex fissures); Grade 4, erosion (4.0 superficial zone delamination; 4.5: mid zone excavation); Grade 5, denudation (5.0: bone surface intact; 5.5: reparative tissue surface present); Grade 6, deformation (6.0: joint margin osteophytes; 6.5: joint margin and central osteophytes) (Figure 2). The total cartilage damage scores for each of the 3 operated groups (O5M, O6M, and O9M) were summed. In each operated group, the OOCHAS scores in each knee condyle, including the medial femoral condyle (MFC), lateral femoral condyle (LFC), medial tibial condyle (MTC), and lateral tibial condyle (LTC), were calculated. To assess the intracondylar subregional changes, the total OOCHAS scores in the anterior, central, and posterior parts in each condyle and all four condyles were summed. In addition, subregional changes in the OOCHAS scores in each operated groups were analyzed.

### 2.6. Biochemical Biomarkers Measurement

The levels of serum CTX-II was quantified using the CartiLaps enzyme-linked immunosorbent assay (ELISA) (Nordic Bioscience, Denmark) according to the manufacturer's instructions. Serum Preclinical CartiLaps ELISA is based on the binding of two identical monoclonal antibodies to cross-linked serum fragments of type II collagen. Briefly, a biotinylated monoclonal antibody was first added to the streptavidin-coated wells of the microtiter plate and then incubated at room temperature for 30 minutes on a mixing apparatus. After washing, the standards, controls, serum samples, and incubation buffer were pipetted into the wells and further incubated at room temperature for 60 minutes. Subsequently, the wells were washed and a solution of peroxidase-conjugated monoclonal antibody was added. After the third washing step, a chromogenic substrate was added into all of the wells and incubated for 15 minutes in complete darkness at room temperature. Finally, the color reaction was terminated using sulfuric acid. The absorbance was measured within 2 hours at 450 nm, and 650 nm was used as a reference.

The levels of serum OC were also measured by ELISA kits (Takara Bio Inc., Japan) using a monoclonal antibody that was specific to undercarboxylated OC (Glu-OC), which was based on the sandwich method. Standards and samples were added into the appropriate wells and incubated for 2 hours at room temperature. After removing the sample solution and washing the wells 3 times, an anti-OC labeled with peroxidase (POD) conjugate solution was added into wells and incubated at room temperature for 1 hour. Next, the sample solution was removed and the wells were washed 4 times. Subsequently, the substrate solution was pipetted into each well and incubated at room temperature for 15 minutes. Finally, the reaction was terminated using sulfuric acid. The absorbance was read immediately at 450 nm using a plate reader.

### 2.7. Statistical and Data Analyses

The data were presented as the mean ± standard error. The Kruskal-Wallis test was adopted to test the differences of the OOCHAS scores between the MFC, LFC, MTC, and LTC in each operated group, the scores between the anterior, central, and posterior subregions in each condyle, the total scores of 3 subregions of all 4 knee condyles, and the scores between the 3 subregions in each operated group. If the Kruskal-Wallis test showed significant differences, and then the Mann-Whitney *U* test with Bonferroni correction was employed for further analysis. The levels of serum CTX-II and OC were also compared between the animals in the 3 operated groups and the animals of the control group of the same ages (group O5M versus group N5M, group O6M versusgroup N6M, and group O9M versus group N9M) using the Mann-Whitney *U* test. Statistical analysis was performed using SYSTAT software (SPSS, version 11.0, Chicago, IL, USA) and *P* < 0.05 was considered statistically significant.

To evaluate the effect of age-related cartilage turnover and concurrent osteoarthritic related cartilage damage on the levels of serum CTX-II and OC, we proposed to assess the summed effect by dividing the mean CTX-II (or OC) levels in osteoarthritic groups (O5M, O6M, and O9M) by the mean CTX-II (or OC) levels, which corresponded to normal control groups (N5M, N6M, and N9M), respectively, which is known as the osteoarthritic-to-normal ratio (ONR). An ONR > 1 indicated elevated serum CTX-II or OC levels that were ascribed to surgical osteoarthritis, which outweighed the effect of normal cartilage turnover. In addition, according to the total OOCHAS scores, the severities of osteoarthritis of the operated rabbits were categorized as 4 groups: mild group (scores = 0.5–10), moderate group (scores = 10.5–20), advanced group (scores = 20.5–30), and far-advanced group (scores > 30). Values of the CTX-II and OC ONRs in these 4 groups and their relationships to the severities of osteoarthritis were evaluated.

## 3. Results

### 3.1. Joint Changes after Surgically Induced Osteoarthritis

**Figure 3:**
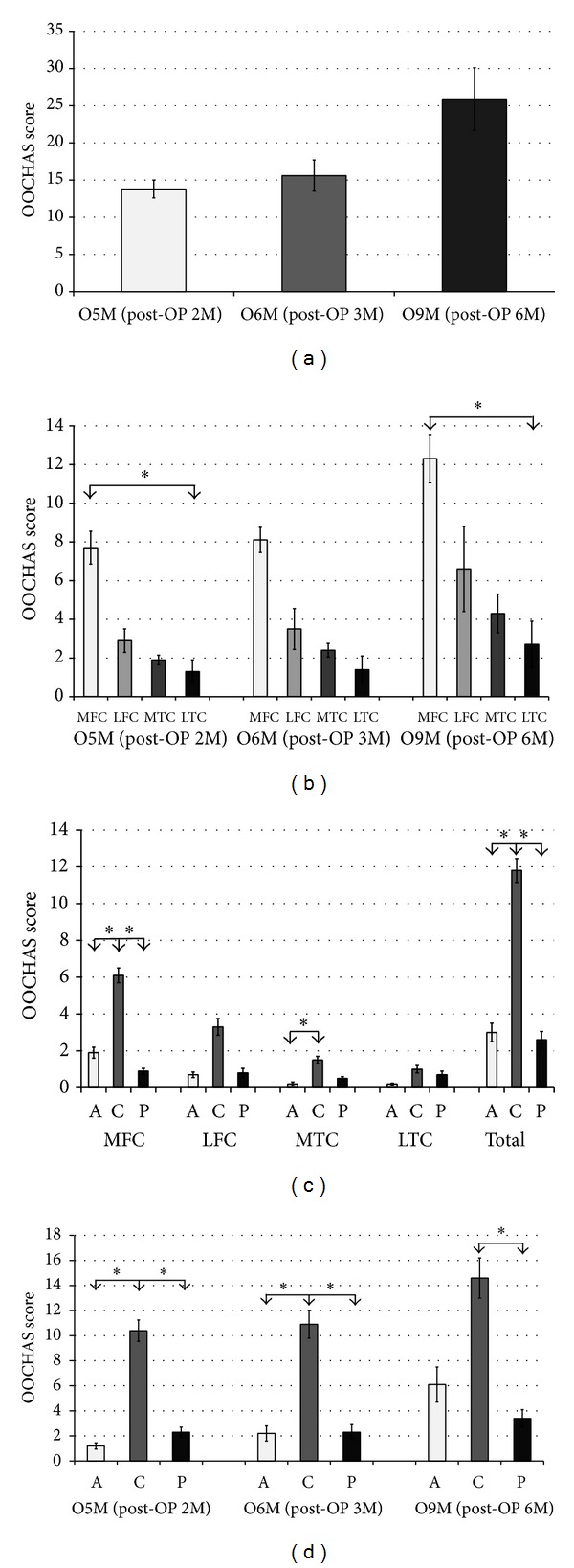
(a) OARSI Osteoarthritis Cartilage Histopathology Assessment System (OOCHAS) scores in operated 5-month-old (O5M), 6-month-old (O6M), and 9-month-old (O9M) rabbits. (b) The OOCHAS scores in the medial femoral condyle (MFC), lateral femoral condyle (LFC), medial tibial condyle (MTC), and lateral tibial condyle (LTC) at each time point. (c) The OOCHAS scores in the anterior (A), central (C), and posterior (P) subregions of each condyle and total knee. (d) The OOCHAS scores in each subregion at each time point. *Significant difference as assessed using the Mann-Whitney *U* tests with Bonferroni correction.

Progressive increases in total OOCHAS scores were found among the operated animals at 2, 3, and 6 months after combined unilateral anterior cruciate ligament transection and partial medial meniscectomy in 3-month-old young rabbits, indicating progressive cartilage damage over time (Figure 3(a)). In all three operated groups, among the 4 condyles of the operated knee, the MFC harbored the most severe osteoarthritic changes, followed by the LFC, MTC, and LTC (Figure 3(b)). Within each knee condyle, the central subregion demonstrated the most severe cartilage damage. The OOCHAS score in the central subregion of the MFC and total score of the central subregions in all 4 condyles were significantly higher than those of the anterior and posterior subregions, respectively (all *P* < 0.001). The score in the central MTC was also significantly higher than the anterior but not significantly different from the posterior MTC. Contrastingly, there were no significant differences in the scores in the subregions of the LFC and LTC (Figure 3(c)). In groups O5M and O6M, the scores in the central subregions were significantly higher than those in the anterior and posterior subregions (*P* = 0.004–0.009). However, in group O9M, the significant difference was only observed between the central and posterior subregions (*P* = 0.015) (Figure 3(d)).

### 3.2. Longitudinal Changes in Serum CTX-II and OC in Normal Controls

**Figure 4:**
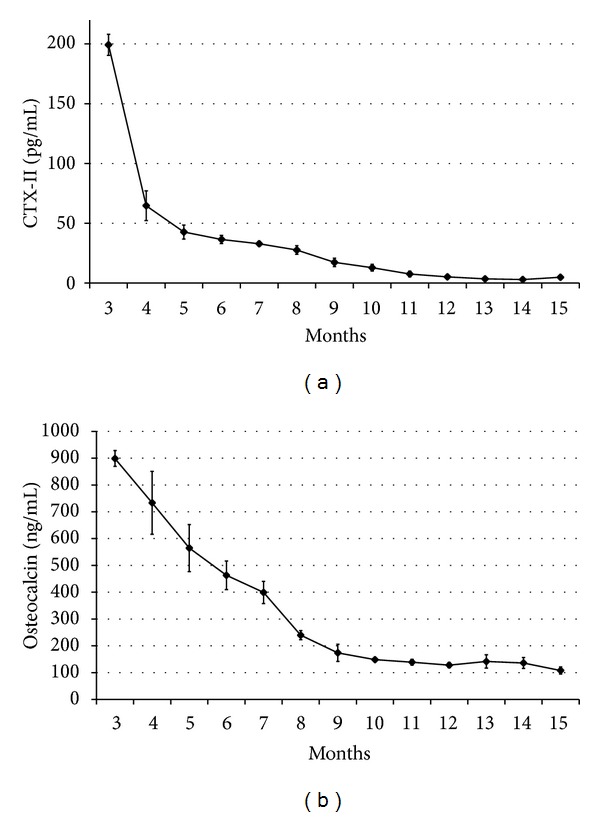
Evolution of serum levels of C-telopeptide of type II collagen (CTX-II) (a) and osteocalcin (OC) (b) in growing rabbits from the age of 3 to 15 months.

The levels of serum CTX-II in the control group decreased markedly from 3 to 5 months and then decreased slowly from 6 to 11 months. Next, the serum CTX-II level remained at a stable low level until month 15 (Figure 4(a)). However, the levels of serum OC in the healthy rabbits decreased almost linearly from 3 to 11 months and were sustained at a stable low level until month 15 (Figure 4(b)).

### 3.3. Cross-Sectional Changes of Serum CTX-II and OC in Operated Groups

**Figure 5:**
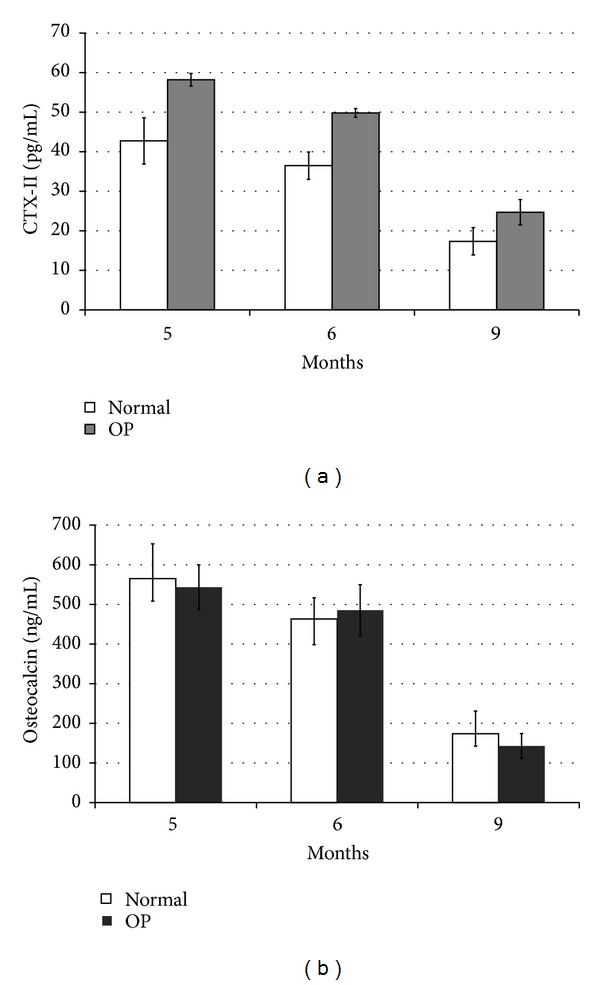
Serum levels of C-telopeptide of type II collagen (CTX-II) (a) and osteocalcin (OC) (b) in operated rabbits and controls. No significant differences were observed in all comparisons.

Although the mean levels of serum CTX-II in all the animals of all 3 operated groups at different time points were higher than animals of the same age in the control group, the differences were not statistically significant (*P* = 0.171–0.352). However, similar to the controls, there was a trend of decreased levels of serum CTX-II at 2, 3, and 6 months after surgically induced osteoarthritis observed among the 3 operated groups (Figure 5(a)). In addition, our results also revealed no significant differences in the mean levels of serum OC in the 3 operated groups compared with the controls (*P* = 0.762–0.914) (Figure 5(b)).

### 3.4. ONRs and Severities of Osteoarthritis

**Figure 6:**
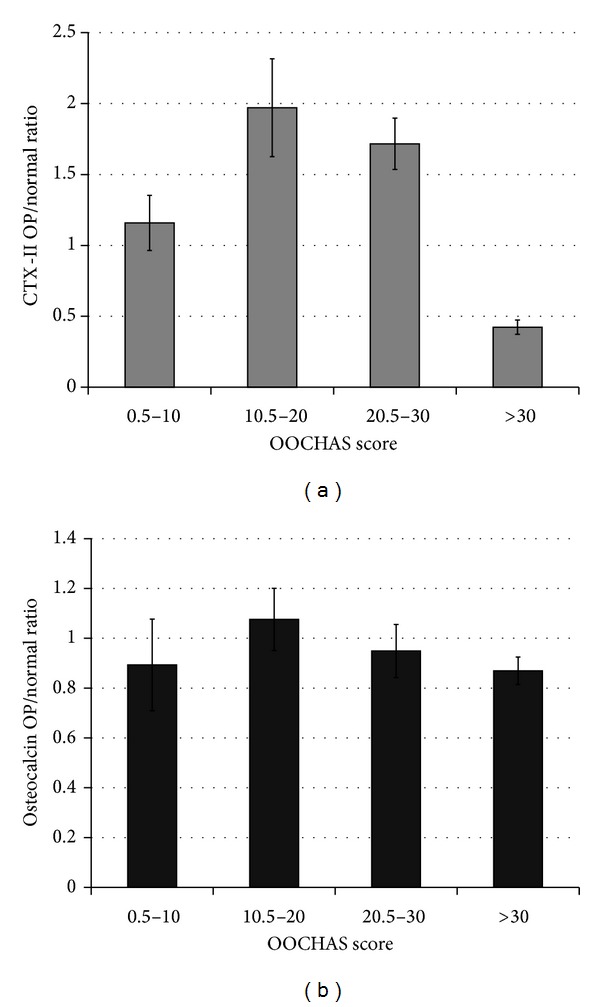
Variation of osteoarthritis-to-normal ratios (ONRs) of CTX-II (C-telopeptide of type II collagen) (a) and OC (osteocalcin) (b) in operated rabbits with different severities of osteoarthritis as categorized according to the OOCHAS scores as: mild (scores = 0–10), moderate (scores = 10.5–20), advanced (scores = 20.5–30), and far-advanced (scores = 30).

The CTX-II ONRs in the operated rabbits with moderate to advanced osteoarthritic damages of articular cartilage were at least 1.5 times higher compared to the controls. However, the mild osteoarthritic group exhibited mildly elevated ONR. Contrastingly, far-advanced osteoarthritis resulted in low ONR (<0.5), which potentially contributed to a robust loss of articular cartilage (Figure 6(a)). However, only a small range of the OC ONRs (0.87–1.08) was found among the operated rabbits with variable severities of osteoarthritis (Figure 6(b)).

## 4. Discussion

Surgical induction of instability in a load-bearing joint in animals can result in cartilage degradation mimicking osteoarthritis. In contrast to humans who may avoid using the injured parts, animals continue to use the traumatized limbs, resulting in a rapid disease progression and providing a good model to study the pathogenesis and pathology of osteoarthritis, a slowly progressive human disease [28]. In the present study, a combined anterior cruciate ligament transection and partial medial meniscectomy method was applied in the right knee to induce sufficient osteoarthritic changes in the young rabbits [24, 25]. This model was successful to induce discernable osteoarthritis in the knees in a short period and progressive cartilage damage over time, although the young rabbits exhibit a higher compensatory capacity of the cartilages compared to adult animals after trauma [24, 29]. Similar to the situation in humans, where approximately 75% of the normal load passes through the medial aspect of the knee [28, 30], the MFCs were the most severely affected after surgically induced osteoarthritis, indicating predominate medial load-bearing in the knees of young rabbits. Moreover, it can be envisaged that the central subregion with maximum kinematic load endured significantly greater cartilage damage than the anterior and posterior subregions early after surgery. In addition, the lack of significant differences of damage between the central and anterior subregions was observed after 6 months, suggesting sequential shifting of kinematic load and cartilage damage from the central to anterior and eventually posterior subregions during the development of surgically induced osteoarthritis.

CTX-II and OC have been proposed as feasible biomarkers for early detection and monitoring of arthritic diseases, and their concentrations reflect processes that are directly implicated in the synthesis or degradation of articular cartilage and bone mineralization, which enables an assessment over shorter periods of time compared to conventional radiography [7–11, 16–24]. Good correlation of the levels of CTX-II to the severity of knee cartilage defects on MRI in subjects with predominantly normal findings on radiographs has also been reported [14]. Furthermore, in an ovariectomized rat model, cartilage matrix breakdown during the early stages of osteoarthritis and a positive correlation between urinary CTX-II levels and histological scores have been demonstrated [31]. However, the CTX-II level may vary considerably with age, gender, hormone replacement therapy, menopause status, and body mass index in humans [20, 32]. In humans, high concentrations of CTX-II levels in children to young adults (20 to 25 years) have been described, and stable levels were observed in adults between 30 and 50 years [20, 33]. High CTX-II levels and cartilage turnover were observed in young rats until 5 to 7 months of age [31, 34].

The effects of age-related cartilage turnover on the CTX-II and OC levels in growing rabbits have not been well addressed. Duclos et al. performed a 5-month longitudinal study using an anterior cruciate ligament transection surgical model in young rabbits and found high CTX-II levels in 2-month old rabbits (approximately 20-fold comparatively to adults) with decreasing levels over the duration of their study [24]. Consistent with Duclos et al., our results confirmed that serum CTX-II in growing rabbits decreased markedly from 3 to 5 months of age. This finding may be interpreted as the articular cartilage of the knee of New Zealand rabbits reaches structural maturity at the time of their sexual maturity (3-4 months) [35]. In addition, in the present study, we deliberately extended the study period for up to one year in order to fully explore the entire spectrum of age-related changes of CTX-II and OC in young to adult rabbits. After the initial dramatic drop, the serum CTX-II levels in the growing rabbits continued to decrease slowly from 6 to 11 months and then remained in a stable low level until 15 months of age. Our results supported the hypothesis that the growth plate activities in the growing rabbits are continuous but slow down gradually after structural cartilage maturity [24, 35]. Moreover, this study highlights that age-related cartilage turnover could continue even up to 11 months of age. Furthermore, promoted bone formation, as reflected in higher baseline levels of OC, may protect against cartilage loss with decreased CTX-II levels having been reported [17]. Contrastingly, the present study demonstrated a gradual and stable decrease in serum OC levels in rabbits from month 5 to month 11, thereafter, similar to CTX-II levels. This finding was suggestive of a parallel correlation between cartilage and subchondral bone formation in the growing rabbits after knee cartilage maturation. Such a correlation may be ascribed to less and less subchondral bone being remodeled from the cartilage synthesized from the growth plates [20].

As reported by Duclos et al., there were positive correlations between serum CTX-II levels and the histomorphological scores of the medial condyle of the knee in adult rabbits but not in young rabbits. Similarly, we found no significant differences in the serum CTX-II and OC levels between growing rabbits with and without surgically induced osteoarthritis. Although choosing adult animals for preclinical studies of osteoarthritis was advised [24], such a selection carries several shortcomings, including a longer animal rearing period, higher cost, the presence of preexisting joint diseases in adult rabbits, and the need for consideration of concurrent aging changes in long duration studies. In the present study, we proposed to use ONR for the overall assessment of osteoarthritis and age-related cartilage turnover in the growing rabbits. Simply, ONR > 1 indicated that the elevated biomarker level ascribed to surgically induced osteoarthritis out-weighed the effect of normal cartilage turnover. Our data supported that elevated CTX-II ONRs were observed in growing rabbits with mild to advanced osteoarthritis. However, for those with far-advanced osteoarthritis and lower CTX-II levels, CTX-II ONR < 0.5 typified, denuded, or tremendously destroyed articular cartilage. Contrastingly, the OC ONRs remained unhelpful in assessing osteoarthritic growing rabbits.

In conclusion, the present study demonstrated that the medial femoral condyles, particularly the central parts, harbored the most severe osteoarthritic changes in growing rabbits following surgically induced osteoarthritis. The serum levels of CTX-II and OC in growing rabbits decreased from month 3 to month 11 and remained stable thereafter. ONR enabled an overall assessment of osteoarthritis and age-related cartilage turnover. Elevated CTX-II ONRs were observed in growing rabbits with mild to advanced osteoarthritis.
